# Expression of Neuroendocrine Markers in Different Molecular Subtypes of Breast Carcinoma

**DOI:** 10.1155/2014/408459

**Published:** 2014-02-19

**Authors:** David L. Wachter, Arndt Hartmann, Matthias W. Beckmann, Peter A. Fasching, Alexander Hein, Christian M. Bayer, Abbas Agaimy

**Affiliations:** ^1^Institute of Pathology, University Hospital, Krankenhausstr 8–10, Erlangen 91054, Germany; ^2^Department of Gynecology and Obstetrics, University Hospital, Universitätsstr 21, Erlangen 91054, Germany

## Abstract

*Background*. Carcinomas of the breast with neuroendocrine features are incorporated in the World Health Organization classification since 2003 and include well-differentiated neuroendocrine tumors, poorly differentiated neuroendocrine carcinomas/small cell carcinomas, and invasive breast carcinomas with neuroendocrine differentiation. Neuroendocrine differentiation is known to be more common in certain low-grade histologic special types and has been shown to mainly cluster to the molecular (intrinsic) luminal A subtype. *Methods*. We analyzed the frequency of neuroendocrine differentiation in different molecular subtypes of breast carcinomas of no histologic special type using immunohistochemical stains with specific neuroendocrine markers (chromogranin A and synaptophysin). *Results*. We found neuroendocrine differentiation in 20% of luminal B-like carcinomas using current WHO criteria (at least 50% of tumor cells positive for synaptophysin or chromogranin A). In contrast, no neuroendocrine differentiation was seen in luminal A-like, HER2 amplified and triple-negative carcinomas. Breast carcinomas with neuroendocrine differentiation presented with advanced stage disease and showed aggressive behavior. *Conclusions*. We conclude that neuroendocrine differentiation is more common than assumed in poorly differentiated luminal B-like carcinomas. Use of specific neuroendocrine markers is thus encouraged in this subtype to enhance detection of neuroendocrine differentiation and hence characterize the biological and therapeutic relevance of this finding in future studies.

## 1. Introduction

Breast carcinoma is the most common cancer of women and generally exhibits a favorable overall 5-year prognosis [1]. However it is becoming increasingly evident that carcinoma of the breast represents a heterogeneous disease with different prognostic subtypes. Invasive mammary carcinomas with neuroendocrine differentiation were first described in 1963 [2] and recognized as a subtype of mammary carcinoma and included in the World Health Organization (WHO) classification in 2003 [3]. According to the current WHO they are defined as carcinomas exhibiting expression of synaptophysin and/or chromogranin A with the exception of small cell carcinoma which is defined morphologically and usually only exhibits limited or less frequent expression of specific neuroendocrine markers but expresses NSE more frequently [3]. The current WHO classification of tumors subdivides carcinomas of the breast with neuroendocrine features into well-differentiated neuroendocrine tumors (NET), poorly differentiated neuroendocrine carcinomas/small cell carcinomas, and invasive breast carcinomas with neuroendocrine differentiation, stating an overall incidence of <1% of all breast carcinomas [3]. However, the true incidence of mammary neuroendocrine neoplasms is difficult to assess because neuroendocrine markers are not routinely used in the diagnostic immunohistochemical panel of breast cancer. It is assumed that neuroendocrine differentiation can be detected more frequently in invasive carcinoma of no special type (NST) and certain morphological special types, particularly cellular invasive mucinous carcinoma and solid papillary carcinoma [3].

Apart from the general histological classification of invasive breast carcinomas, Perou et al. defined molecular subtypes: luminal A, luminal B, HER2 enriched, and basal-like carcinomas using DNA microarray technology in a set of invasive breast carcinomas of no histologic special type [4]. This classification has since then been shown to be of prognostic [5] and predictive [6] value with luminal A carcinomas showing the best prognosis. It is now known that some histologic special types of invasive breast carcinoma cluster to mainly one molecular subtype: Weigelt et al. showed that cellular invasive mucinous carcinoma and neuroendocrine carcinomas mostly represent the luminal A molecular subtype [7].

In a case-control study Wei et al. described a worse prognosis of breast carcinomas with neuroendocrine differentiation when compared to carcinomas of no histologic special type [8]. We recently encountered several metastatic breast carcinomas which exhibited diffuse neuroendocrine differentiation associated with high-grade histologic features (falling short of criteria used to diagnose small cell carcinoma of the breast [9]) but at the same time retaining estrogen receptor expression. These data can result in considerable confusion regarding treatment options and prognostic impact when clinicians are confronted with the diagnosis of invasive breast cancer with neuroendocrine differentiation. In particular, it is still unclear whether to treat poorly differentiated breast carcinoma with neuroendocrine differentiation analogous to poorly differentiated invasive breast cancer or similar to poorly differentiated neuroendocrine carcinomas of other sites. The aim of the current study is to examine the frequency and extent of neuroendocrine differentiation in immunohistochemically characterized different molecular subtypes of breast carcinomas using a wide array of immunohistochemical markers.

## 2. Material and Methods

Invasive breast carcinomas of different molecular subtypes diagnosed between 2002 and 2009 at the Institute of Pathology of the University Hospital Erlangen, Germany, were retrieved from routine surgical pathology files by a computer assisted search. Molecular subtypes were determined using immunohistochemical surrogate markers, which were stained during routine surgical pathology practice. Luminal carcinomas were defined by nuclear estrogen receptor expression in more than 10% of tumor cells. Furthermore luminal carcinomas were divided in luminal A and luminal B carcinomas using a Ki67 index cut-off value of 13% as proposed by Cheang et al. [10] To enhance segregation of luminal A and B carcinomas for the purpose of this study, we included in the luminal A group only grade 1 carcinomas and in the luminal B group only grade 3 carcinomas. Since differentiation of luminal A and luminal B carcinomas is not entirely reliable on morphologic and immunohistochemical grounds, we use the terms luminal A-like and luminal B-like in this study. The HER2 amplified group was defined by lacking expression of the estrogen and progesterone receptors and strong circular membranous staining for HER2 in more than 10% of tumor cells (DAKO score 3+). The basal-like subtype was defined as triple negative.

Using these criteria, we arbitrarily selected 30 luminal A-like carcinomas (including 10 cases of invasive lobular carcinomas, ILC), 20 luminal B-like carcinomas, 20 HER2 amplified carcinomas and 30 basal-like carcinomas without further knowledge of the histological features of the carcinomas (apart from the ILC no other histologic special types were included). The different study groups were filled consecutively from a large cohort of breast carcinomas at our institution until at least 20 unselected cases were present in every subgroup. To identify possible precursor lesions of invasive neuroendocrine carcinoma, we also included 10 cases of low-grade and 10 cases of high-grade ductal carcinoma in situ (DCIS) and 80 normal breast tissue specimens. From these cases a tissue microarray (TMA) was constructed: representative areas of the lesions (or the normal tissue) were marked on the glass slides and a tissue core with a diameter of 2 mm was punched out of the donor block and transferred onto the recipient block. The recipient block was then cut (3 *μ*m) and the sections mounted on SuperFrost slides (Menzel Gläser, Braunschweig, Germany). All slides were stained with haematoxylin and eosin (H&E).

To determine neuroendocrine differentiation we used antibodies against chromogranin A (monoclonal mouse anti-human antibody, clone LK2H10, Beckman Coulter Inc., Diagnostics Division Headquarters, 250 South Kraemer Boulevard, Brea CA 92821-6232, USA, dilution 1 : 500) and synaptophysin (monoclonal mouse anti-human antibody, clone Snp88, Biogenex Laboratories Inc., 4600 Norris Canyon Road, San Ramon, CA 94583, USA, dilution 1 : 50). Further, we assessed the expression of some other non-specific markers known to be frequently expressed in neuroendocrine neoplasms including CD56 (monoclonal mouse anti-human antibody, clone 1B6, Novocastra Laboratories Inc., Balliol Business Park West, Benton Lane, NE12 8EW Newcastle Upon Tyne, UK, dilution 1 : 50), CD117 (polyclonal rabbit anti-human, code A4502, Dako Denmark, Produktionsvej 42, DK-2600 Glostrup Denmark, dilution 1 : 100) and NSE (monoclonal mouse anti-human antibody, clone BBS/NC/VI-H14, Dako, dilution 1 : 300). Immunohistochemical stainings were performed on 1 *μ*m slides using the fully automated slide preparation system “Benchmark XT System” (Ventana Medical Systems Inc., 1910 Innovation Park Drive, Tucson, Arizona, USA). H&E and immunohistochemical stainings were evaluated by two of the authors (DLW, AA) and the extent of positive staining (%) and staining intensity (negative, weak, moderate, and strong) were noted. Only moderate to strong immunohistochemical staining was considered positive. Unfortunately the WHO does not provide a clear cut-off to define neuroendocrine differentiation in breast carcinomas. Since the WHO cites a study by Sapino et al. who used a cut-off of 50% [11] and Wei et al. confirmed prognostic impact of neuroendocrine differentiation using the same cut-off, we also regarded a minimum of 50% of tumor cells with moderate to strong expression of chromogranin A or synaptophysin as indicative of neuroendocrine differentiation. After identification of the carcinomas with neuroendocrine differentiation using the TMA slides the whole sections of these cases were examined for assessment of morphological features of the carcinomas and the adjacent tissue.

## 3. Results (Table 1)

### 3.1. Normal Breast Tissue

**Figure 1:**
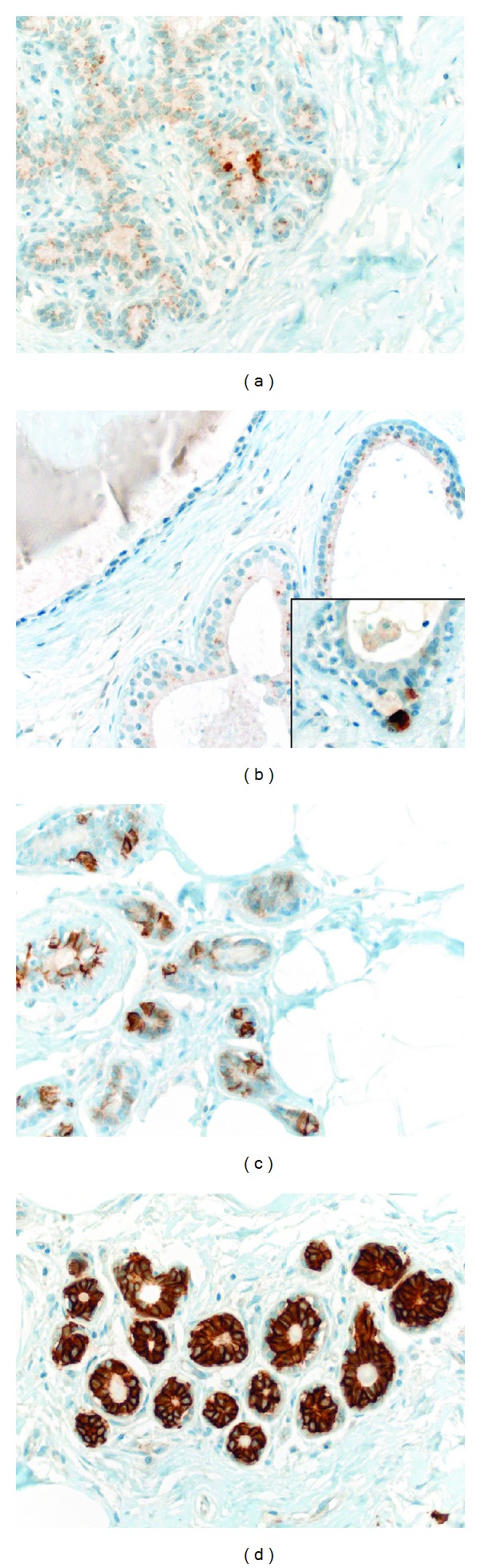
Expression of specific (synaptophysin & chromogranin A) and non-specific (CD56 & CD117) neuroendocrine markers in normal breast tissue. (a) Expression of synaptophysin in luminal cells of normal breast tissue. (b) Synaptophysin expression in probably apocrine differentiated luminal cells (inset: strongly synaptophysin positive basal cell, consistent with a true neuroendocrine cell; however, no chromogranin A expression was seen in this area). (c) CD56 expression in a subset of normal luminal cells. (d) CD117 expression in the majority of luminal cells in normal breast tissue.

Of the 69 evaluable normal tissue cores none showed chromogranin A positive cells and only 5 (7.2%) cases revealed moderate to strong apical expression of synaptophysin in isolated mostly luminal cells (Figure 1(a)). Some of these luminal cells showed probable apocrine differentiation (Figure 1(b)). One case showed a single positive basally located cell (Figure 1(b) inset), possibly representing a true neuroendocrine cell, but no chromogranin A expression was seen. Regarding the non-specific neuroendocrine markers, 44 of 62 normal tissues (70.9%) showed moderate to strong expression of CD56 in up to 70% (usually around 30%) of luminal cells (Figure 1(c)) and the vast majority of the normal breast tissues (55 of 67; 82.1%) showed moderate to strong diffuse expression of CD117 in the luminal cells (usually more than 80% of luminal cells, Figure 1(d)). 25 of the 64 (39.1%) normal tissues showed positivity for NSE in the luminal cells.

### 3.2. Low-Grade DCIS

All of the 6 available sections were negative for chromogranin A, synaptophysin and all of 7 available cases were negative for CD56. One of 5 samples showed focal expression of CD117 and 4 of 5 cases revealed diffuse expression of NSE.

### 3.3. High-Grade DCIS

**Figure 2:**
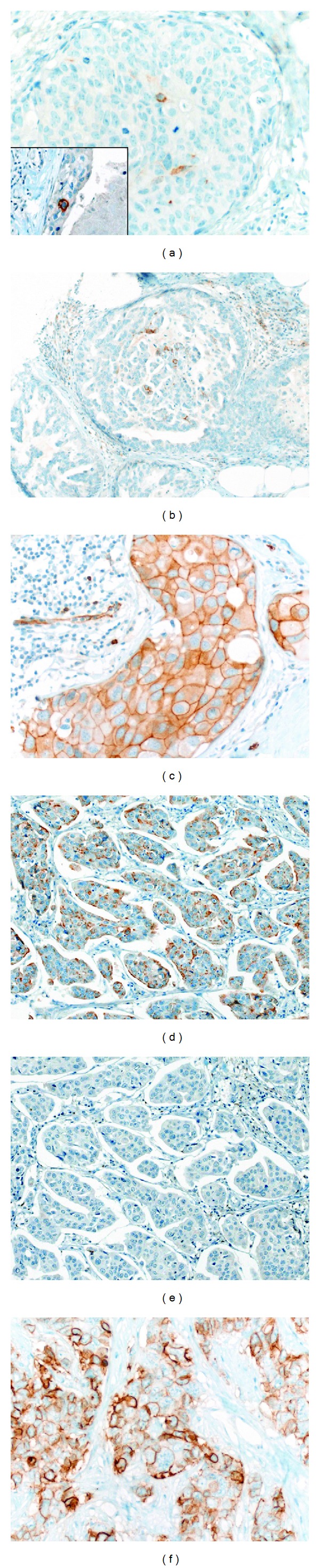
Expression of specific (synaptophysin and chromogranin A) and non-specific (CD56, CD117) neuroendocrine markers in DCIS and invasive carcinoma. (a) Isolated chromogranin A positive cells in high-grade DCIS (inset: isolated synaptophysin positive cells in the same case). (b) High-grade DCIS with isolated CD56 positive cells. (c) High-grade DCIS diffusely expressing CD117. (d) Luminal B carcinoma NST diffusely expressing chromogranin A. (e) The same luminal B carcinoma is negative for synaptophysin. (f) Diffuse expression of CD56 in a triple-negative carcinoma.

One of 10 cases showed strong but only focal expression of chromogranin A (Figure 2(a)) and 2 of 10 cases revealed focal expression of synaptophysin (Figure 2(a) inset). One of 10 cases showed moderate focal (<10% of tumor cells) expression of CD56 (Figure 2(b)) and 1 of 10 cases showed moderate diffuse (>80%) expression of CD117 (Figure 2(c)) with another 2 cases showing moderate focal (<10%) expression of CD117. 3 of 10 cases showed diffuse moderate to strong expression of NSE and another 3 of 10 cases showed moderate to strong focal expression of NSE.

### 3.4. Luminal A-Like Carcinomas

Chromogranin A was negative in all of the 25 cases (including 9 invasive lobular carcinomas). Synaptophysin was focally expressed in 1/24 carcinomas, notably this positive case was an ILC with moderate staining in <10% of tumor cells. CD56 was diffusely (>70% of tumor cells) expressed in 3/24 (12.5%) cases, including 2 of 8 (25%) ILC. One additional case showed focal strong expression in 30% of tumor cells. CD117 was diffusely positive in 1 of 22 (4.5%) carcinomas. This case was an ILC (1/7, 14.3%) with moderate staining in more than 80% of tumor cells. NSE was diffusely expressed in 17 of 23 (73.9%) of cases with moderate to strong staining in at least 60% of the tumor cells and moderate focal expression in <10% of tumor cells in 1 case. According to WHO criteria, no carcinomas with neuroendocrine differentiation were found.

### 3.5. Luminal B-Like Carcinomas

Of 18 stained cases 2 (11.1%) showed moderate to strong diffuse expression of chromogranin A in over 80% of tumor cells (Figure 2(d)). One additional case showed focal moderate granular cytoplasmic expression in 20% of tumor cells. One of the chromogranin A positive tumors was negative for synaptophysin (Figure 2(e)). 3 cases (16.7%) showed diffuse staining (one case with staining in 70% of tumor cells, the other two with staining in more than 90% of tumor cells) for synaptophysin. Another 3 of 18 cases showed moderate to strong but only focal staining. One of the diffusely synaptophysin positive tumors was negative for chromogranin A. According to WHO criteria, neuroendocrine differentiation was found in 4 cases (22.2%).

All of the 18 tested cases were negative for CD56. One of 18 (5.6%) cases showed strong diffuse CD117 staining in >80% of tumor cells and another one revealed moderate focal CD117 staining in <10% of tumor cells. Of 17 tested carcinomas, 6 showed moderate to strong diffuse NSE positivity in more than 80% of tumor cells, another 6 cases showed only focal expression in less than 50% of tumor cells.

### 3.6. HER2 Amplified Carcinomas

One of 15 stained HER2 amplified carcinomas showed strong focal chromogranin A expression in only <1% of tumor cells and two of the 15 stained cases showed moderate to strong focal synaptophysin staining in <1% and <10% of tumor cells respectively. All of the 14 tested carcinomas were negative for CD56 and 2 of 15 cases (13.3%) showed moderate diffuse CD117 expression in >80% of tumor cells. 2 of 15 (13.3%) cases showed diffuse moderate NSE expression and another 4 cases only showed focal expression. No carcinomas with specific neuroendocrine differentiation were found.

### 3.7. Basal-Like Carcinomas

All of the 29 tested carcinomas were negative for chromogranin A and one of the 29 cases stained moderately and focally (<10% of tumor cells) for synaptophysin. 4/29 (13.8%) cases showed moderate to strong diffuse CD56 expression in >80% of tumor cells (Figure 2(f)) and another 4 cases showed moderate to strong focal expression in less than 50% of tumor cells. 12 of the 29 (41.4%) carcinomas showed moderate to strong diffuse expression of CD117 in >80% of tumor cells. 13 of 29 (44.8%) cases revealed moderate to strong diffuse NSE expression in >80% of tumor cells. No carcinomas with specific neuroendocrine differentiation were found.

Taken together, 4 of 18 (22.2%) stained cases of luminal B-like carcinomas exhibited neuroendocrine differentiation using current WHO criteria. The luminal A-like, HER2 amplified and basal-like carcinoma subgroups as well as all the DCIS cases did not reveal diffuse neuroendocrine differentiation. No clear-cut neuroendocrine cell progenitor lesion was seen in the normal tissues.

### 3.8. Clinicopathological Features of Breast Carcinomas with Neuroendocrine Differentiation (Table 2)

Three of the cases affected patients around 80 years of age and showed lymphovascular invasion and/or lymph node metastases at presentation. The fourth case without lymph node metastases presumably originated in ectopic axillary mammary tissue and relapsed locally after 2 years. Two of the four patients died of disease after 15 and 36 months, respectively; the other two were lost to follow-up.

**Figure 3:**
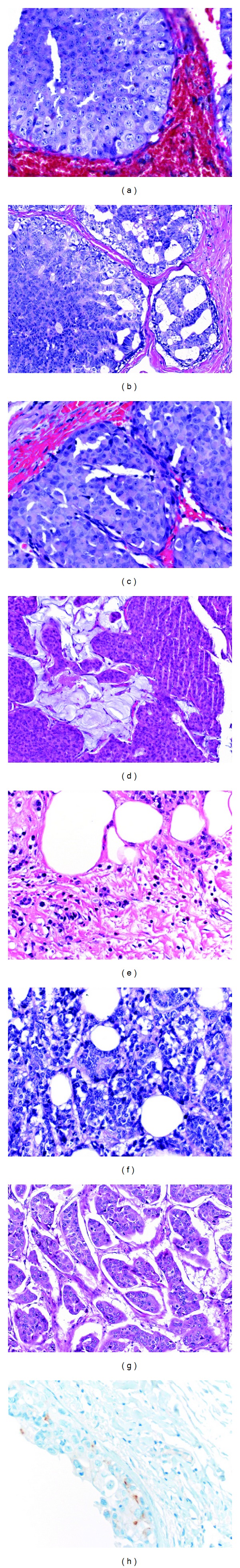
Morphological features of luminal B carcinomas with neuroendocrine differentiation. (a) Solid (insular) formations of tumor cells. (b) Cribriform, DCIS-like areas of the same case. (c) Solid infiltrates of plasmacytoid tumor cells with subnuclear eosinophilic cytoplasm. (d) Same case with focal mucinous differentiation. (e) Infiltrative border of a carcinoma with scattered tumor cells. (f) The same case with focal rosette-like structures. (g) Prominent peritumoral clefts, imparting a micropapillary-like morphology, (h) The same case with perifocal high-grade DCIS with only focal expression of chromogranin A.

Morphologically the four carcinomas revealed a wide spectrum of architectural and cytological features: the first case showed solid (insular) infiltrates of medium-sized tumor cells with bright cytoplasm, coarse nuclear chromatin, and multiple prominent nucleoli (Figure 3(a)) and in other areas pseudocribriform DCIS-like structures (Figure 3(b)). Another case was composed of solidly arranged medium-sized tumor cells with sometimes plasmacytoid morphology and perinuclear condensation of eosinophilic cytoplasm. The nuclei displayed fine chromatin and prominent nucleoli. At the periphery of the small insular solid structures some of the tumor cells showed subnuclear eosinophilic cytoplasmic granules (Figure 3(c)). Of note small areas comprising <10% of the tumor area showed extracellular mucin pools (Figure 3(d)). The third case showed a solid trabecular growth pattern with isolated scattered tumor cells at the infiltrative border (Figure 3(e)). Focally poorly formed small rosettes were seen (Figure 3(f)). In one slide a minute focus of high-grade DCIS (solid type with necrosis) was appreciated but was not available on deeper sections for immunohistochemical stainings. The fourth case revealed a carcinoma composed of medium-sized tumor cells with eosinophilic cytoplasm and vesicular nuclei with fine chromatin and prominent nuclei. The tumor cells grew in small solid formations with prominent shrinking artifacts imparting an invasive micropapillary-like pattern (Figure 3(g)). Perifocal high-grade DCIS with solid, cribriform and micropapillary architecture, and central necrosis was seen. Immunohistochemically >80% of the invasive tumor cells showed moderate granular chromogranin A expression and showed moderate membranous expression of CD117, whereas synaptophysin, NSE, and CD56 were negative. Only <10% of the cells of the high-grade DCIS showed moderate granular chromogranin A expression (Figure 3(h)) and >80% of the DCIS cells membraneously expressed CD117.

The adjacent normal tissue in all of these cases showed no signs (morphologically or immunohistochemically) of neuroendocrine cell hyperplasia.

## 4. Discussion

In our experience, tumors resembling NET of the gastrointestinal tract or small cell carcinomas of the breast are extremely rare. On the other hand, identifying carcinomas with neuroendocrine differentiation lacking the features of NET, small cell carcinoma, cellular mucinous carcinoma, or solid papillary carcinoma using morphology alone can be very challenging or even impossible. We have recently encountered several cases of aggressive metastatic breast cancers, which showed features of neuroendocrine differentiation with preserved nuclear estrogen receptor expression. These cases were not recognized as having neuroendocrine differentiation in the primary breast tumors during routine workup. The lacking histomorphological features of neuroendocrine differentiation in many such cases and the lack of standardized immunohistochemical testing for neuroendocrine markers in invasive breast carcinomas suggest underestimation of neuroendocrine differentiation in invasive breast carcinomas, especially in tumors of the molecular luminal B subgroup.

Given that the neuroendocrine differentiation often cannot be diagnosed on morphologic grounds alone, routine testing for neuroendocrine markers should be considered in the future. In this context, it is of note that two of the four carcinomas with neuroendocrine differentiation in the present study only expressed chromogranin A or synaptophysin and not both of these markers, arguing for a routine immunohistochemical panel including both chromogranin A and synaptophysin.

Additionally our findings show that CD56, CD117, and NSE, although relatively sensitive in neuroendocrine neoplasms at other sites, should not be used as markers of neuroendocrine differentiation in the breast as they have a very low specificity and are also expressed in a high frequency in normal breast tissue. Interestingly CD56 is expressed in a considerable subset of luminal A-like and basal-like carcinomas although the significance (if any) of this finding remains unknown. CD117 was expressed in about 40% of the basal-like carcinomas, in around 13% of HER2 amplified carcinomas and in 5% of luminal carcinomas making this marker of limited or no value in segregating neuroendocrine subtypes of breast carcinoma.

In the present study, we were not able to identify clear-cut neuroendocrine cells in normal breast tissue adjacent to carcinomas with neuroendocrine differentiation and tissues unrelated to these cases. Recently, Kawasaki and colleagues demonstrated isolated and increased (hyperplastic) neuroendocrine cells in normal appearing breast tissue adjacent to DCIS with neuroendocrine differentiation [12]. In the 3 reported cases, the DCIS showed diffuse neuroendocrine differentiation and the patients were 28 to 38 years old. The authors suggested that the DCIS arose from these hyperplastic neuroendocrine cells. If true, this points to at least two differing pathways of development of neuroendocrine neoplasia in the breast. In the first model, neoplasia could develop in a hyperplasia-neoplasia sequence similar to some types of pulmonary and gastrointestinal counterparts [12]. In the second model, neuroendocrine differentiation could represent a secondary phenomenon by tumor cell evolution through ongoing mutations and subsequent clonal overgrowth of the neuroendocrine tumor cells in established carcinoma [13, 14]. Given that the accompanying DCIS of the luminal B-like carcinomas NST with neuroendocrine differentiation in this study only showed focal neuroendocrine differentiation and the adjacent normal tissue did not show neuroendocrine cell hyperplasia, we hypothesize that in these cases the neuroendocrine cell population developed secondarily. The large size of the tumors, lack of progenitor lesions, and sometimes patchy staining pattern of neuroendocrine markers in whole-section slides support this hypothesis. This mode of development would seem analogous to the well-studied mixed-adenoneuroendocrine carcinomas (MANEC) of the gastrointestinal tract [15] and other sites which also show only partial neuroendocrine differentiation, potentially posing diagnostic difficulties in limited biopsy material. However molecular data regarding these types of tumors of the breast are lacking.

Confrontation with a diagnosis of invasive breast cancer with neuroendocrine differentiation often results in confusion of the clinician and the patient regarding prognosis or therapeutic options. This underlines the need to specify the subtype and grade of neuroendocrine tumor as accurately as possible. As cellular invasive mucinous carcinomas (with low-grade morphology) clusters in the luminal A molecular subgroup [7] and solid papillary carcinoma is considered to mostly represent an intraductal carcinoma with possible associated invasive carcinoma [16], these tumors obviously represent low-grade carcinomas with a very good prognosis. On the other hand small cell carcinoma of the breast is a neuroendocrine carcinoma of high-grade, although in a recent study it has been shown to be less aggressive than, for example, small cell carcinoma of the lung [9]. Carcinomas NST (as in our study) with neuroendocrine differentiation probably represent the most frequent invasive breast carcinomas with neuroendocrine differentiation. In a recent study Wei and colleagues showed that if WHO 2003 criteria are applied (leaving out solid papillary carcinoma and only including 3 cellular invasive mucinous carcinomas) and cases of carcinomas with neuroendocrine differentiation are matched with control patients (carcinomas NST without neuroendocrine differentiation), the carcinomas with neuroendocrine differentiation carry a worse prognosis than those without [8].

As the vast majority of carcinomas with neuroendocrine differentiation seem to cluster in the intrinsic luminal subgroups with preserved nuclear estrogen receptor expression antihormonal treatment is a reasonable treatment option. In the present study all of the carcinomas with neuroendocrine differentiation were found in the luminal B-like subgroup. These carcinomas have been shown to be more aggressive with poorer prognosis and better response to chemotherapy compared to luminal A carcinomas so that chemotherapy is to be considered in the appropriate clinical setting. However, although modern prognostic and predictive biomarkers are emerging [17], it is known that currently only a subgroup of patients benefits from chemotherapy and that response to therapy cannot be reliably predicted in individual patients [18]. Additional biomarkers for prediction are therefore necessary and diagnosis of neuroendocrine differentiation might become a therapeutic option in the future. Thus, larger studies are needed to assess the predictive value of routine testing for neuroendocrine differentiation in breast carcinomas. In the future even special targeted therapies might be an option for this patient subgroup as isolated case reports have described promising results using octreotide in this setting [19].

In conclusion, we showed that neuroendocrine (trans)differentiation is a frequent event in luminal B-like breast carcinomas. The routine search for neuroendocrine differentiation in invasive breast carcinoma apart from cellular invasive mucinous carcinoma, solid papillary carcinoma, and small cell carcinoma should focus on luminal B-like carcinomas in order to understand the significance of this phenomenon in the future. As these carcinomas often present with advanced stage disease, studies exploiting the neuroendocrine differentiation with targeted therapies might be of benefit.

## Figures and Tables

**Table 1:** Expression of specific (chromogranin A and synaptophysin) and non-specific (CD56, CD117, NSE) neuroendocrine markers in different molecular subtypes of breast carcinoma, DCIS, and normal tissue.

Molecular subtype	Chromogranin A	Synaptophysin	CD56	CD117	NSE
Luminal A					
Focal (<50%)	0/25 (0%)	1/24 (4.2%)	1/24 (4.2%)	0/22 (0%)	1/23 (4.3%)
Diffuse (>50%)	0/25 (0%)	0/24 (0%)	3/24 (12.5%)	1/22 (4.5%)	17/23 (73.9%)
Luminal B					
Focal (<50%)	1/18 (5.6%)	3/18 (16.7%)	0/18 (0%)	1/18 (5.6%)	6/17 (35.3%)
Diffuse (>50%)	**2/18 (11.1%)**	**3/18 (16.7%)**	0/18 (0%)	1/18 (5.6%)	6/17 (35.3%)
HER2					
Focal (<50%)	1/15 (6.7%)	2/15 (13.3%)	0/14 (0%)	0/15 (0%)	4/15 (26.7%)
Diffuse (>50%)	0/15 (0%)	0/15 (0%)	0/14 (0%)	2/15 (13.3%)	2/15 (13.3%)
Basal-like					
Focal (<50%)	0/29 (0%)	1/29 (3.4%)	4/29 (13.8%)	0/29 (0%)	0/29 (0%)
Diffuse (>50%)	0/29 (0%)	0/29 (0%)	4/29 (13.8%)	12/29 (41.4%)	13/29 (44.8%)
High-grade DCIS					
Focal (<50%)	1/10 (10%)	2/10 (20%)	1/10 (10%)	2/10 (20%)	3/10 (30%)
Diffuse (>50%)	0/10 (0%)	0/10 (0%)	0/10 (0%)	1/10 (10%)	3/10 (30%)
Low-grade DCIS					
Focal (<50%)	0/6 (0%)	0/6 (0%)	0/7 (0%)	1/5 (20%)	1/5 (20%)
Diffuse (>50%)	0/6 (0%)	0/6 (0%)	0/7 (0%)	0/5 (0%)	4/5 (80%)
Normal tissue					
Focal (<50%)	0/69 (0%)	5/69 (7.2%)	44/62 (70.9%)	10/67 (14.9%)	25/64 (39.1%)
Diffuse (>50%)	0/69 (0%)	0/69 (0%)	1/62 (1.6%)	55/67 (82.1%)	25/64 (39.1%)

**Table 2:** Clinicopathologic features at presentation and follow-up data.

Case	Age	Tumor size (mm)	Multifocality (number of tumors)	Lymphovascular invasion	Metastases	Follow-up
1	59	30	No	No	Bone	Local relapse after 2 years; died of disease after 36 months

2	77	35; 5	Yes (2)	Yes	Axillary lymph nodes	Disease free after 3 years and then lost to follow-up

3	83	27	No	No	Axillary lymph nodes	Disease free after 3 months and then lost to follow-up

4	85	35; 7; 5	Yes (3)	Yes	Axillary lymph nodes	Skin metastases after 4 months; died of disease after 15 months
